# Evaluating Emotional Intelligence in Medical Students: A Comparative Analysis of Their Trait Emotional Intelligence (TEI) Scores Before and During the COVID-19 Pandemic

**DOI:** 10.7759/cureus.74335

**Published:** 2024-11-24

**Authors:** Shilpi Bhat, Swati Mittal, Smriti Sinha, Ritvija Dixit

**Affiliations:** 1 Physiology, School of Medical Sciences and Research, Greater Noida, IND; 2 Physiology, Vallabhbhai Patel Chest Institute, New Delhi, IND; 3 Physiology, Mamta Academy of Medical Sciences, Hyderabad, IND; 4 Physiology, Amrita School of Medicine, Faridabad, IND

**Keywords:** emotional intelligence, medical education, medical students, pandemic, questionnaire

## Abstract

Introduction: Medical education, which generally relied heavily on intelligence quotients, has found a new value in emotional intelligence (EI), specifically after the challenges faced during the COVID-19 pandemic. The COVID-19 pandemic has had some apparent and some intangible effects, as this required an emergency switch to online teaching and learning.

Aim: This study was conducted to compare the trait emotional intelligence (TEI) score of medical undergraduates with the increasing number of years of medical curriculum. The COVID-19 pandemic serendipitously provided an opportunity to compare the TEI scores in pre-pandemic and during pandemic times.

Materials and methods: Trait emotional intelligence was computed using a pre-validated Trait Emotional Intelligence Questionnaire-Short Form (TEIQue-SF). The study was conducted in two time frames and in two study groups, the Class of 2019 (pre-pandemic cohort) and the Class of 2020 (pandemic cohort). Further, the pre-pandemic cohort’s EI score was assessed twice, initially in August 2019 (Timeframe 1 (T1) which was before the pandemic) and secondly in October 2020 (Timeframe 2 (T2) which was during the pandemic) in Phase II and the COVID-19 first wave was peaking in India. The TEI scores of the pre-pandemic cohort and pandemic cohort were compared using an unpaired T-test. The pre-pandemic cohort scores assessed in two time frames were compared using a paired T-test.

Results: The pandemic cohort showed significantly less total TEI (p < 0.05) and well-being score (p < 0.05) in the pre-pandemic cohort from August 2019 (T1) to October 2020 (T2) (p = 0.036).

Conclusions: The low TEI scores in the pandemic cohort may be attributed to the sudden psychological and social effects of the pandemic. Moreover, with advancing age and years of medical curriculum, the TEI scores increased.

## Introduction

In light of the constantly evolving medical profession, which emphasizes the importance of holistic patient care, it is pertinent to prepare physicians who can undertake the stressors of high-quality healthcare while managing their emotions simultaneously. Need of the hour is an effective amalgamation of emotions and cognition to promote qualitative healthcare [1]. This brings us to the concept of emotional intelligence (EI), broadly defined as a set of skills that facilitates self-awareness, understanding, and management of how our emotions affect ourselves, others, and our performance [2]. Trait emotional intelligence (TEI), a subtype of EI, precisely refers to emotion-related self-perceptions and dispositions that can significantly contribute to resilience in stressful situations [3]. Given the high demands of quality and productivity in healthcare coupled with the rising prevalence of mental illness among medical students, EI seems to be vital for both academic success and mental health protection [4,5]. Training in EI among healthcare workers is purported to improve leadership qualities and enhance communication skills, with improvements in patient satisfaction and compliance at the same time preventing burnout [6]. The recognition of the importance of EI in the development of well-rounded, empathetic future physicians has prompted the National Medical Council (NMC) to consider its integration into the medical curriculum in the form of Attitude Ethics Communication (AETCOM) modules.

As the world has started coming back to normalcy, the enigma called COVID-19 is still being unravelled, and its impact and effects are still being pondered. The learnings from this catastrophe may pave the way for a well-prepared future. The pandemic had a far-reaching impact on the lives of medical students globally, with rising anxiety and depression reported due to heightened uncertainty, modified curriculums, curtailed social interactions, and perplexity regarding their choice of profession [7,8]. Thus, it seems prudent to analyze the TEI and the impact that COVID-19 had on the TEI of medical students to identify potential protective factors that may help mitigate the effects of any such future pandemics on EI. The effect of the pandemic on the TEI can only be commented on if studies are done to evaluate the TEI of the same cohort during pre-pandemic as well as during the pandemic times. Such longitudinal studies, to the best of our knowledge, have yet not been conducted on Indian medical graduates, who provide a major working healthcare force globally.

Based on the aforementioned concepts, this study was initially designed to determine the impact of medical curriculum on TEI in a cohort of newly joined medical students. However, the unexpected advent of COVID-19 provided the researchers with a unique opportunity to analyze the effect of the pandemic on the TEI of medical students and to compare it with the non-pandemic cohort of medical students.

## Materials and methods

Study design and setting

**Figure 1:**
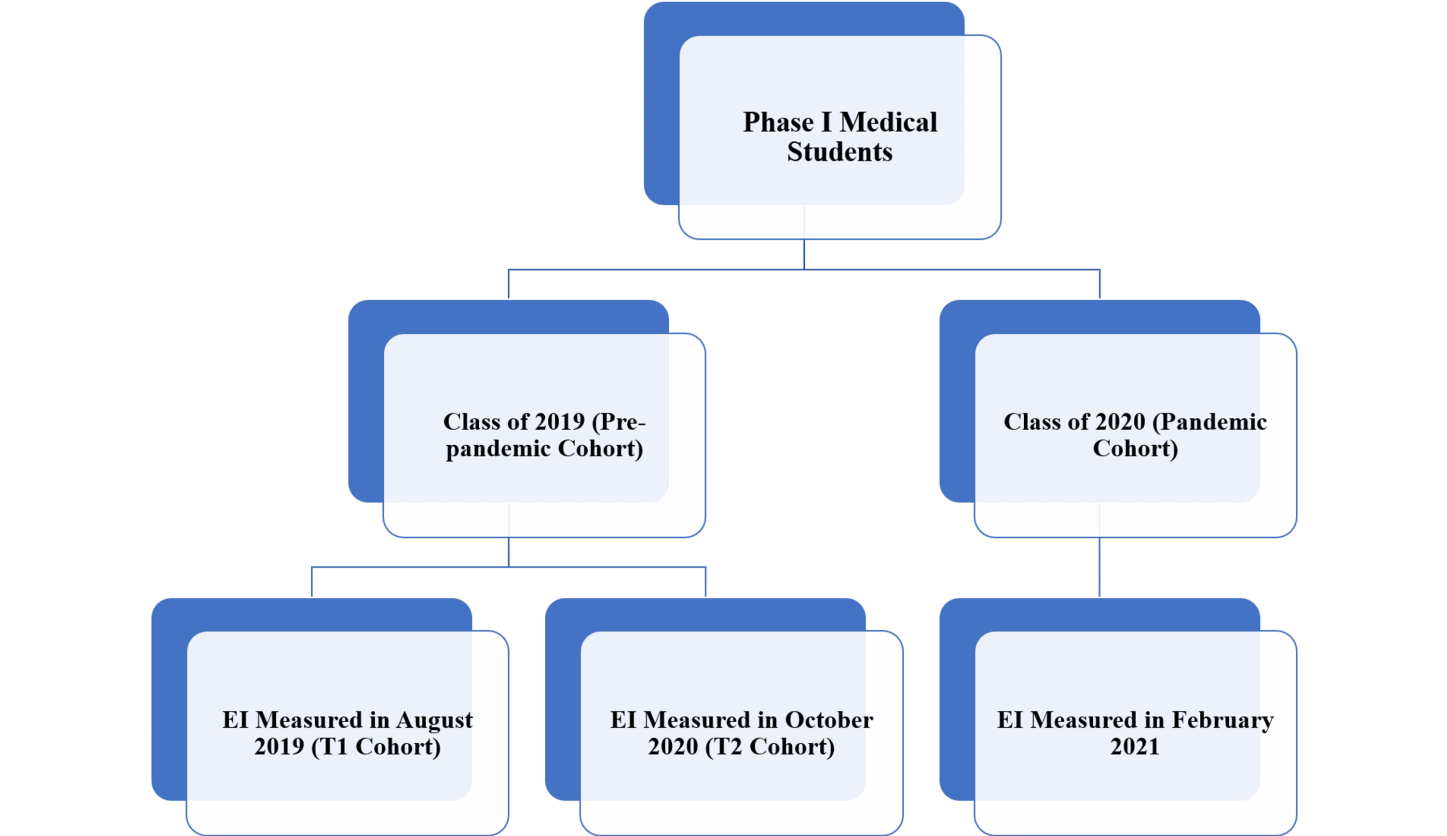
Grouping of medical students and various timeframes of the assessment of trait emotional intelligence EI: emotional intelligence; T1: Timeframe 1; T2: Timeframe 2

**Figure 2:**
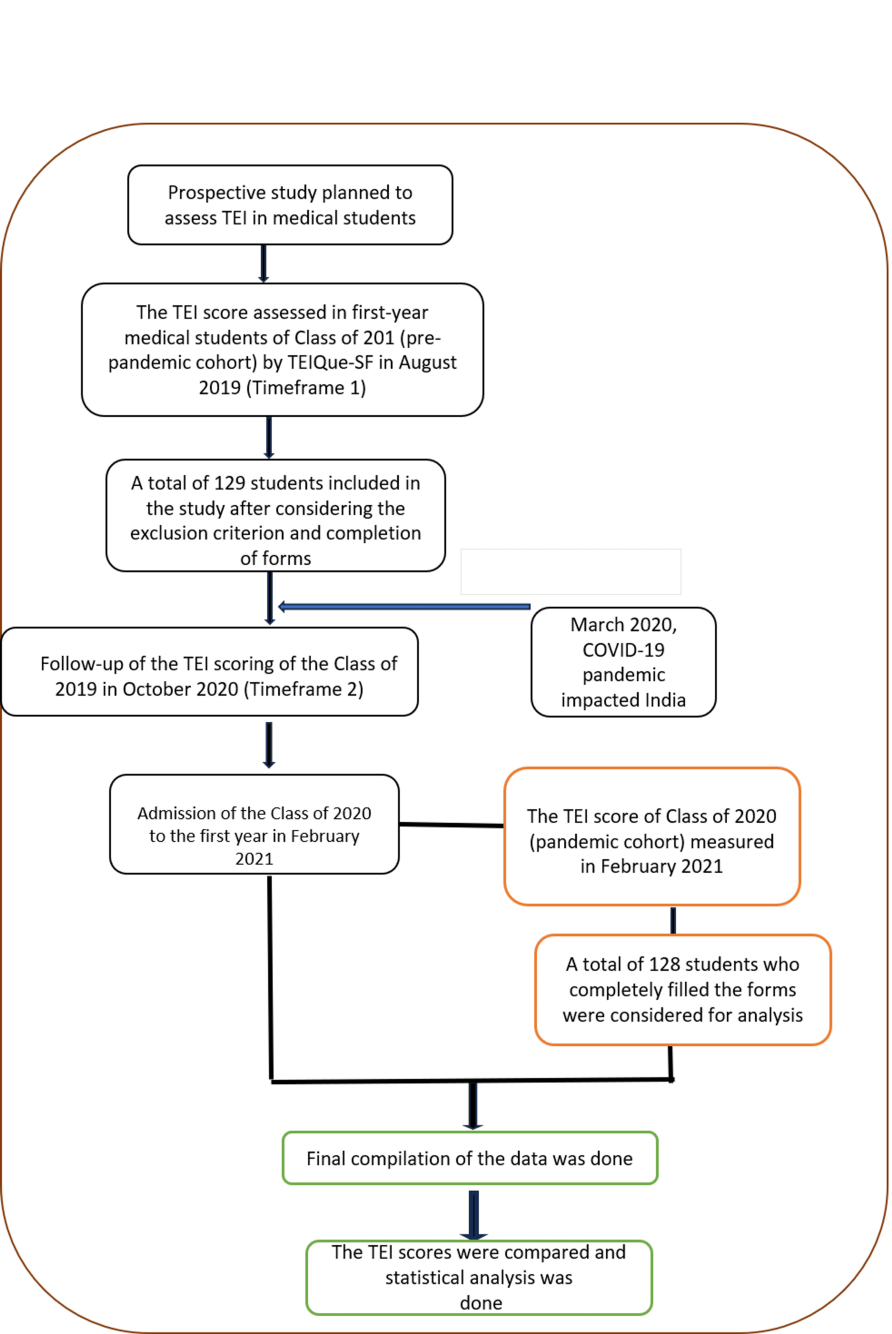
Flowchart of the study EI: emotional intelligence; TEI: trait emotional intelligence; TEIQue-SF: Trait Emotional Intelligence Questionnaire – Short Form

This was a longitudinal observational, electronically distributed questionnaire-based study conducted from August 2019 to March 2021 at the School of Medical Sciences and Research, a medical college in Greater Noida, India. The study was initiated after obtaining approval from the institutional ethics committee (approval number: SU/SMS&R/76-A/2019/186). To determine the impact of the medical curriculum on Phase I medical students (Class of 2019, the pre-pandemic cohort), the TEI score was assessed twice, once at the time of joining and again after completion of one year. To assess the impact of the pandemic on TEI, the scores of the pre-pandemic (Class of 2019) and pandemic (Class of 2020) cohorts of medical students were compared. The grouping of students and the flowchart of the study are depicted in Figures 1-2, respectively.

Sample size

The institute admits 150 medical students for undergraduate medical education. The sample size was based on convenience sampling. Medical Phase I students of the Class of 2019 were informed about the nature, type, and implications of the study during a physical classroom session. All students who provided consent and were willing to participate formed the study sample. Students having a known history of drug abuse, chronic illness, or psychological issues and who did not give consent were excluded from the study.

Data collection procedure 

Pre-pandemic Phase

The participants of the Class of 2019 were emailed a Google link (Google Inc., Mountain View, CA) of the questionnaire in their personal e-mails in August 2019 (Timeframe 1 (T1)). The questionnaire consisted of two sections; section one included items regarding socio-demographic variables, while section two assessed their baseline TEI through a pre-validated Trait Emotional Intelligence Questionnaire-Short Form (TEIQue-SF) questionnaire (Appendix A). The participants were supposed to fill out and submit the questionnaire within one month of joining the medical course. Out of the 145 forms circulated, 135 responses were received, out of which six forms were excluded based on the exclusion criteria. The next TEI score was planned to be collected after a year into the medical course to assess the impact of the curriculum's rigour and the participants' age.

Pandemic Phase: Class of 2019

As the pandemic struck India around March 2020 severe restrictions, including lockdown, were implemented. It provided an unexpected real-life opportunity to evaluate the probable effect of the pandemic on the TEI. The TEI score of the pandemic cohort was reassessed as per the initial plan again in October 2020 (Timeframe 2 (T2)). The participants were sent the TEIQue-SF online questionnaire after approximately one year of the initial assessment. This time coincided with the peak of the first wave of the COVID-19 pandemic in India. The T2 assessment of TEI could potentially point towards the impact of the pandemic on the cohort apart from the effects of the medical curriculum. 

Pandemic phase: Class of 2020

In order to gain a deeper understanding of the impact of the pandemic on the TEI of medical students, with other conditions being much the same apart from the pandemic, the baseline TEI scores of the newly joined medical students of the Class of 2020 (pandemic cohort) were planned. The pandemic cohort joined the medical curriculum slightly delayed due to COVID restrictions in February 2021, following the peak of the first COVID wave in August in India. The procedure for data collection and assessment of TEI for the pre-pandemic cohort was closely repeated for the pandemic cohort too. Out of the 146 forms circulated, 133 responses were received, while five forms were excluded based on the exclusion criteria.

Study tool

The TEI of the participants was assessed by the TEIQue-SF questionnaire by Petrides and Furham (2009), which is a 30-item scale designed to measure global TEI and is based on the long form of the TEIQue [9]. The scale consists of a combination of both negatively and positively worded items and is a promising research tool for TEI assessment due to its brevity, evidence of its predictive validity, and good basic psychometric properties for student samples in numerous countries. The reliability of the TEIQue-SF scale is calculated as 0.816. The participants were asked to self-rate the questionnaire on a seven-point Likert-type scale from one (completely disagree) to seven (completely agree). Scores were calculated for each of the four facets of well-being: emotionality, sociability, and self-control, and a total EI score was derived for each participant based on the criteria mentioned.

Statistical analysis

The data were collated and analyzed statistically using IBM SPSS Statistics software for Windows, version 28 (IBM Corp., Armonk, NY), and a p-value of <0.05 was considered statistically significant. The continuous variable was expressed as means and standard deviation. Statistical analysis comprised of the Student’s paired t-test for comparing the change in baseline and pandemic TEI scores of the pre-pandemic cohort (Class of 2019). The Student’s unpaired t-test was used to analyze the change in baseline TEI scores between the pre-pandemic (Class of 2019) and pandemic cohort (Class of 2020). The results were tabulated and are presented for ease of understanding.

## Results

A total of 129 students participated in the study from the Class of 2019 (pre-pandemic cohort), while 128 participated from the Class of 2020 (pandemic cohort). The mean age of the participants has been depicted in Table 1. The pandemic group had a significant age difference as compared to pre-pandemic due to the delay in their joining attributed to the pandemic.

**Table 1:** Comparison of mean age between the pre-pandemic group at Timeframe 1 and the pandemic group p: probability value by unpaired t-test; *p<0.05 is considered statistically significant

Variable	Pre-pandemic group (N=129 at Timeframe 1)	Pandemic group (N=128)	p-value	T stat value
Mean age (years)	19.38 +/- 0.98	20.31+/- 0.91	0.0007*	3.779

The majority of the participants in both the pre-pandemic as well as pandemic groups were females, accounting for 82 (63.5%) in the pre-pandemic group while slightly higher count 90 (70.3%) among the pandemic group. The pre-pandemic group showed a higher baseline total TEI score, and the difference in comparison to the pandemic group was found to be statistically significant. The mean total TEI score of males was found to be higher as compared to females in both the pre-pandemic and pandemic groups, but the gender-wise comparison was not found to be statistically significant as depicted in Table 2. 

**Table 2:** Comparison of the baseline total TEI scores of the pre-pandemic and pandemic groups p: probability value by unpaired t-test; SD: standard deviation; p<0.05 considered statistically significant; TEI: trait emotional intelligence

Gender	Number of participants		Total TEI scores (Mean±SD)		p-value	T value
	Pre-pandemic group (N=129)	Pandemic group (N=128)	Pre-pandemic group (scores in Timeframe 1)	Pandemic group		
Male	47 (36.4%)	38 (29.7%)	4.7±0.93	4.3±0.71	0.240	0.675
Female	82 (63.6%)	90 (70.3%)	4.4±0.20	4.0±0.47	0.203	1.366
Total	129 (100%)	128 (100%)	4.39±0.55	4.12±0.55	0.043*	2.210

The total TEI and the well-being and self-control facets of the pre-pandemic group were higher than the pandemic group, and the difference was recorded to be statistically significant as depicted in Table 3. Contrarily, the emotionality facet was found to be higher among the pandemic group as compared to the pre-pandemic group, but the difference was not found to be significant.

**Table 3:** Comparison of the four facets of the baseline TEI scores between the pre-pandemic and pandemic group p: probability value by unpaired t-test; SD: standard deviation; p<0.05 considered statistically significant; TEI: trait emotional intelligence; EI: emotional intelligence

S.no	Variables	Total TEI and each subset of TEI scores (Mean±SD)			p-value	T value
		Pre-pandemic group (N=129 TimeFrame 1)	Pandemic group (N=128)			
1.	Total EI score	4.39 +/- 0.55	4.12+/- 0.55		0.043*	2.210
2.	Well-being	5.36 +/- 0.77	4.77 +/- 0.91		0.003*	3.038
3.	Emotionality	4.67 +/- 0.79	4.75 +/- 0.76		0.517	-0.441
4.	Sociability	4.38 +/- 0.81	4.32 +/- 0.68		0.820	0.339
5.	Self-control	4.27 +/- 0.77	3.86 +/- 0.76	0.042*		2.340

To assess the effect of the pandemic on the TEI, the TEI scores of the pre-pandemic group were assessed in two time frames, T1 (the baseline score assessed in year 2019) and T2 (assessed in year 2020, after the pandemic). The total TEI score showed a statistically significant increase after the pandemic as compared to its own baseline score. None of the other subsets of TEI seem to display any significant change in the two assessment time frames, as highlighted in Table 4.

**Table 4:** Comparison of Total and four facets of TEI scores of pre-pandemic group during different time frames. p: probability value by unpaired t-test; SD: standard deviation; p<0.05 considered statistically significant; TEI: trait emotional intelligence; EI: emotional intelligence

S.no	Variables	Total TEI and each subset of TEI score of the pre-pandemic group (Mean±SD, N=129)		p-value	T value
		Time frame T1 (Baseline)	Time frame -T2 (After one year)		
1.	Total EI score	4.39 +/- 0.55	4.66 +/- 0.65	0.036*	-1.899
2.	Well-being	5.36 +/- 0.77	5.14 +/- 0.78	0.222	1.251
3.	Emotionality	4.67 +/- 0.79	4.87 +/- 0.74	0.127	-1.576
4.	Sociability	4.38 +/- 0.81	4.28 +/- 0.96	0.952	0.060
5.	Self-control	4.27 +/- 0.77	4.04+/- 1.26	0.562	0.587

Further evaluation of the effect of the COVID-19 pandemic on the TEI of medical students in various age groups/classes during the ongoing pandemic was made, and the results are presented in Table 5. The students of Phase II reported a higher total TEI as compared to the Phase I students, and the difference was highly significant (p˂0.01). Although all the facets of TEI were reported to be higher among Phase II students, the difference was not significant.

**Table 5:** Comparison of the total and four facets of TEI between the pandemic and pre-pandemic groups assessed during pandemic times p: probability value by unpaired t-test; SD: standard deviation; p<0.05 considered statistically significant; TEI: trait emotional intelligence; EI: emotional intelligence

S.no	Variable	Baseline and each subset of the TEI score (Mean±SD)		p-value	T value
		Pandemic group (Class 2020, N=128)	Pre-pandemic group (Class 2019, N=129, Timeframe 2)		
1.	Total EI score	4.12 +/- 0.55	4.66 +/- 0.65	0.0002**	-3.524
2.	Well-being	4.77+/- 0.91	5.14 +/- 0.78	0.053*	-1.664
3.	Emotionality	4.75 +/- 0.76	4.87 +/- 0.74	0.486	-0.614
4.	Sociability	4.32 +/- 0.68	4.28 +/- 0.96	0.620	0.177
5.	Self-control	3.86 +/- 0.76	4.04 +/- 1.26	0.254	-0.664

## Discussion

Emotional intelligence provides the ability to control one's temperament, be more adaptable, and manage oneself, all of which can help one perform better in high-stress professions like healthcare [10]. Epidemics and pandemic outbreaks cause a great deal of emotional distress in the general population, and healthcare providers are no exception to this disruption. 

Comparison of baseline TEI scores among pre-pandemic and pandemic cohorts

This longitudinal study was conducted to assess the impact of the COVID-19 pandemic on successive batches of medical students’ TEI. It was found that the pandemic cohort had significantly lesser total TEI as well as well-being and self-control scores than the non-pandemic group, even though the age of the pandemic cohort is greater than that of the non-pandemic cohort. Similar outcomes were obtained in a retrospective longitudinal study conducted by Farah-Franco et al. on dental students. They reported that COVID-19 showed a significant decrease in interpersonal and self-perception domains and subscales of happiness and optimism, though an increase in stress tolerance was seen [11,12]. Contrarily, a study on student pharmacist leaders observed the pandemic cohort to have a higher emotional intelligence assessment (EIA) as compared to the non-pandemic cohort, with the scores being significantly higher on the relationship management aspect [13]. This can be attributed to the lockdowns and virtual settings, which underscored the value of emotional intelligence in handling stressful situations.

Comparison of TEI scores of the class of 2019 during two timeframes

According to a study by Baba et al., healthcare workers perceived their EI to be above average during the COVID-19 pandemic [14, 15]. Similar effects echoed in the observations made in our study, where the total EI score of the Class of 2019 was taken in two time frames, T1 (pre-pandemic time) and T2 (during the pandemic), with the T2 score being higher than the T1 score in the same population. This phenomenon could be explained by the fact that an unpredictable crisis usually contributes to a person’s mental and emotional development by triggering behaviour of acceptance and positive reinterpretation regarding the adverse event. The higher scores of the emotionality and sociability subsets of TEI depicted in our study point towards emotional adaptability for the situational crisis named COVID-19. Moreover, the human body and mind cannot operate in full mobilization for an endless amount of time due to energy constraints. As a result, an acute crisis eventually becomes a chronic one if it is not resolved, and there occurs a relative adjustment to the new circumstances [16]. The COVID-19 pandemic allowed the emergence of newer ways of information dissipation, providing an opportunity to be emotionally expressive, influence others emotionally, and have control over one’s emotions.

Comparison of TEI scores in different phases of the pre-pandemic and pandemic cohorts

Even during the pandemic times, Phase II students exhibited a higher total TEI score as compared to Phase I students, supporting the extent of evidence in the literature that TEI increases with age [17,18]. Age leads to emotional stability, and it has been demonstrated that TEI can be utilized in explaining emotional awareness during development [19]. On the contrary, Khraisat et al. showcased EI decline in medical students with progressing medical years [20].

In search of further insights, this study delved into certain key factors of TEI in relation to COVID-19. Threats of infections result in negative emotions that can be contagious and may have ramifications on how people feel and react to others [21]. The pre-pandemic cohort was aptly found to have difficulty restraining their emotions during the pandemic times in comparison to pre-pandemic time, as depicted by the decline in their well-being and self-control scores during the T2 timeframe as compared to their baseline scores. Well-being comprises trait happiness and trait optimism, which were lowest at the time of assessment, as India was just recuperating from the first COVID wave and imposed lockdowns along with increased destabilization and uncertainty. Adverse emotional reactions like anger, frustration, anxiety, and sadness related to the pandemic loomed heavily during this period, and adaptation to this situational crisis and return to a relatively emotionally balanced state requires time [22, 23]. Trait emotional intelligence is a proven effective tool in traversing difficult times, and high EI scorers in our study were likely to reformulate the effects of the pandemic positively without engaging in self-criticism and, at the same time, implement certain coping strategies in their lives to overcome the adversity [4].

Limitations

As EI assessments were made on self-reported measures, there could be biases, especially during stressful times. The shift to online learning and altered social interactions could have impacted the students’ EI in ways that are not directly attributable to the pandemic's emotional toll but rather to the changes in their learning environment. The findings from our study cannot be generalized to institutions with different educational environments. The heightened stress and anxiety associated with the pandemic might temporarily alter EI scores, particularly in areas like stress management, which may not reflect the students' true, long-term EI levels.

Educational significance

The balance between knowledge, technical skills, and interpersonal skills are the building blocks of a healthy doctor-patient relationship. The experience of the pandemic has impacted the EI of medical personnel, potentially influencing their ability to manage patients' emotions effectively. This calls for an increased focus on a holistic approach to medical education globally. Though the ravaging phase of the pandemic has been over, this study highlights the importance of resiliency and the need for monitoring and reinforcing emotional health, especially of medical students, so that the impact of similar situations can be mitigated in the future.

## Conclusions

The COVID-19 pandemic was both a time of tribulation and possibility. This study showed that the levels of TEI were significantly lower in the pandemic as compared to the pre-pandemic cohort. The pre-pandemic cohort revealed heightened TEI scores during the pandemic as compared to their baseline score during non-pandemic times. The study also revealed increased TEI scores among Phase II students as compared to Phase I, reinforcing the fact that age has a positive influence on TEI scores. Medical students, as students and future professionals, form the most vulnerable group to bear the burden of a pandemic, and their ability to cope and bounce back should be assessed and evaluated. A better comprehension of EI dynamics is required to be put forward during medical training to cope with unfamiliar stressful situations. Finally, the advancement in the concept of metacognition will also assist in addressing individual differences and the need for an improved physician-patient relationship.

## Appendices

Appendix A

The TEIQue-SF Questionnaire

**Table 6:** Trait Emotional Intelligence Questionnaire-Short Form Scale of measurement: 1: completely disagree; 7: completely agree Source: [9]

Statements	Responses						
1. Expressing my emotions with words is not a problem for me.	1	2	3	4	5	6	7
2. I often find it difficult to see things from another person’s viewpoint.	1	2	3	4	5	6	7
3. On the whole, I’m a highly motivated person.	1	2	3	4	5	6	7
4. I usually find it difficult to regulate my emotions.	1	2	3	4	5	6	7
5. I generally don’t find life enjoyable.	1	2	3	4	5	6	7
6. I can deal effectively with people.	1	2	3	4	5	6	7
7. I tend to change my mind frequently.	1	2	3	4	5	6	7
8. Many times, I can’t figure out what emotion I'm feeling.	1	2	3	4	5	6	7
9. I feel that I have a number of good qualities.	1	2	3	4	5	6	7
10. I often find it difficult to stand up for my rights.	1	2	3	4	5	6	7
11. I’m usually able to influence the way other people feel.	1	2	3	4	5	6	7
12. On the whole, I have a gloomy perspective on most things.	1	2	3	4	5	6	7
13. Those close to me often complain that I don’t treat them right.	1	2	3	4	5	6	7
14. I often find it difficult to adjust my life according to the circumstances.	1	2	3	4	5	6	7
15. On the whole, I’m able to deal with stress.	1	2	3	4	5	6	7
16. I often find it difficult to show my affection to those close to me.	1	2	3	4	5	6	7
17. I’m normally able to “get into someone’s shoes” and experience their emotions.	1	2	3	4	5	6	7
18. I normally find it difficult to keep myself motivated.	1	2	3	4	5	6	7
19. I’m usually able to find ways to control my emotions when I want to.	1	2	3	4	5	6	7
20. On the whole, I’m pleased with my life.	1	2	3	4	5	6	7
21. I would describe myself as a good negotiator.	1	2	3	4	5	6	7
22. I tend to get involved in things I later wish I could get out of.	1	2	3	4	5	6	7
23. I often pause and think about my feelings.	1	2	3	4	5	6	7
24. I believe I’m full of personal strengths.	1	2	3	4	5	6	7
25. I tend to “back down” even if I know I’m right.	1	2	3	4	5	6	7
26. I don’t seem to have any power at all over other people’s feelings.	1	2	3	4	5	6	7
27. I generally believe that things will work out fine in my life.	1	2	3	4	5	6	7
28. I find it difficult to bond well even with those close to me.	1	2	3	4	5	6	7
29. Generally, I’m able to adapt to new environments.	1	2	3	4	5	6	7
30. Others admire me for being relaxed.	1	2	3	4	5	6	7
